# The use of therapeutic drug monitoring for early identification of vedolizumab response in Saudi Arabian patients with inflammatory bowel disease

**DOI:** 10.1038/s41598-023-28566-4

**Published:** 2023-01-31

**Authors:** Doaa Anbarserry, Mahmoud Mosli, Yousef Qari, Omar Saadah, Rana Bokhary, Ahmed Esmat, Mohammed Alsieni, Ahmed Shaker, Ramu Elango, Sameer Alharthi

**Affiliations:** 1 Department of Pharmacology, Faculty of Medicine, King Abdulaziz University, Jeddah, Saudi Arabia; 2 Department of Medicine, Faculty of Medicine, King Abdulaziz University, Jeddah, Saudi Arabia; 3 Department of Pediatrics, Faculty of Medicine, King Abdulaziz University, Jeddah, Saudi Arabia; 4 Department of Pathology, Faculty of Medicine, King Abdulaziz University, Jeddah, Saudi Arabia; 5 Department of Genetic Medicine, Faculty of Medicine, King Abdulaziz University, Jeddah, Saudi Arabia; 6 Inflammatory Bowel Disease Research Group, King Abdulaziz University, Jeddah, Saudi Arabia; 7 Inflammatory Bowel Disease Unit, King Abdulaziz University Hospital, King Abdulaziz University, Jeddah, Saudi Arabia

**Keywords:** Biomarkers, Diseases, Gastroenterology, Medical research

## Abstract

Vedolizumab is a humanized monoclonal antibody used to treat moderate-to-severe inflammatory bowel disease (IBD). The aim of the study was to assess the effectiveness of the induction of vedolizumab trough level in predicting short-term (week 14) clinical outcomes, and covariates that affect the response in Saudi Arabian patients. This prospective, real-life study included a total of 16 patients (4 Crohn's disease (CD) and 12 ulcerative colitis (UC)) with a confirmed diagnosis of IBD and generally naïve to receiving vedolizumab therapy. Using ELISA assay, vedolizumab induction trough and peak levels were measured at weeks 0, 2, and 6. The follow-up assessment was at week 14, where clinical outcomes were measured using the partial Mayo score for UC, and the CD activity score (CDAI), and Harvey Bradshaw index (HBI) for CD. At week 14, 9 patients (52.9%) out of 16 patients demonstrated response to therapy; clinical remission was reported in 5 patients (29.4%), and in 4 cases a clinical response was noted (23.5%). Clinical remission at week 14 was linked significantly with week 6 median vedolizumab levels in responders (25.1 µg/ml 95% CI: 16.5–42.9) compared to non-responders (7.7 µg/ml, 95% CI: 4.6–10.6) (*P* = 0.002*)*. Receiver operator curve analysis at week 6 identified a cut-off > 8.00 µg/mL for short-term clinical remission. Also, at week 14, BMI significantly correlated with week 6 vedolizumab trough levels (*P* = 0.02). No other covariates correlated with drug levels at any time point examined. Week 6 early vedolizumab trough level measurements in IBD patients predicted short-term week 14 clinical remission.

## Introduction

IBD is a chronic illness generated by an unbalanced autoimmune response to intestinal flora^1^. IBD broadly refers to two types of inflammatory conditions: Crohn’s disease (CD) and ulcerative colitis (UC). IBD management is non-curative and directed mainly at counteracting chronic inflammation. Indeed, achieving sustained clinical remission without serious adverse effects is perhaps the primary goal of IBD management. In recent decades, the focus of IBD treatment has evolved away from traditional medicines like corticosteroids, aminosalicylates, and immunosuppressants to biological medications that target inflammatory pathways^1^. The first pro-inflammatory cytokine targeted by biological drugs was the tumor necrosis factor alpha (TNF-α), which is thought to have an essential role in maintaining chronic inflammation in IBD. Anti-TNF alpha monoclonal antibodies adalimumab (ADA), golimumab (GOL), and infliximab (IFX) are used to treat patients with UC that do not respond to conventional therapies.

Patients with CD that are considered to have high-risk features may benefit from early use of biologics^2^. However, anti-TNF medicines may be limited in terms of their immunogenic potential, leading to decreased responsiveness over time through the formation of anti-drug antibodies (ADAs). Primary non-response (PNR) to anti-TNF agents might be revealed in 10–40% of patients following induction therapy (i.e., at 14 weeks)^3^, while secondary loss of response (LOS) is reported after one year in 24–46% of patients^4^. Failure of anti-TNF therapy and any associated adverse effects have prompted the introduction of other biologics, such as selective leukocyte trafficking inhibitors. The α4β7- Integrin that is present on the outer surface of circulating immune cells, and interacts with MADCAM-1 expressed specifically in the gastrointestinal tract, is considered a therapeutic target in IBD^5^.

Vedolizumab is a monoclonal antibody designed to target α4β7, thereby explicitly acting on the intestine and preventing leukocyte trafficking through impeding the binding of α4β7 with the mucosal vascular addressin cell adhesion molecule—1 (MADCAM-1). Vedolizumab was licensed in 2014 (Takeda Pharmaceuticals International) to treat moderate-to-severe UC and CD in individuals that failed to respond to anti-TNF medication or other conventional treatments. Vedolizumab is administered intravenously, and a 100% bioavailability is anticipated. However, inter- and intra-individual variability in vedolizumab distribution and clearance have been shown^6,7^. Moreover, vedolizumab concentrations in the intestinal tissue and their correlation with serum level were reported^8^.

Three GEMINI randomized controlled clinical trials^9–11^ showed the efficacy of vedolizumab; a number of other studies have shown a favorable safety profile. Moreover, there is increasing evidence supporting the efficacy and safety profile of vedolizumab in real-world studies^12,13^. In the VISIBLE study, a novel subcutaneous (SC) formulation of vedolizumab was used in patients that had a previous intravenous vedolizumab exposure. The trial has proved the efficacy of SC vedolizumab in moderate-to-severe UC patients during the maintenance period^14^.

Even though vedolizumab has proved to be effective and safe in a broad group of individuals with IBD, non-response to this treatment has been reported. Some studies suggest that non-response is lower in bio-naïve patients than those previously exposed to other biologics^8^. Serum therapeutic concentrations have been linked to endoscopic mucosal healing, clinical remission, and clinical response in multiple vedolizumab trials^15–19^. A recent study found that vedolizumab serum levels in combination with vedolizumab tissue levels correlate with clinical outcomes^16^; the recommendation of that study was to use tissue vedolizumab as an additional factor in considering therapeutic drug monitoring approaches during vedolizumab therapy.

The majority of research on the effectiveness and safety of vedolizumab in IBD mainly emerged from Western world, with little research coming from Asian nations. Data specific to Asia are necessary since genetic and environmental variations may affect therapy outcomes and complication rates^20^. These data are extremely inadequate in Saudi Arabia, therefore, in the present study, we investigate whether vedolizumab trough concentrations could be used as an early predictor for short-term response.

## Materials and methods

### Patient population, study design, and outcomes

This was a prospective cohort study involving IBD patients with a confirmed diagnosis following up at King Abdulaziz University Hospital, in Jeddah, Saudi Arabia. Informed consent for participation was obtained from all participants, all patients that started vedolizumab between October 2020 and September 2021 were recruited for the trial. At weeks 0, 2, and 6, patients received vedolizumab intravenous infusion (300 mg) for the induction phase and underwent follow-up assessment at week 14. Before starting vedolizumab, patient demographics and clinical features were obtained. Throughout the induction period, blood samples for vedolizumab trough level analysis were drawn prior to vedolizumab infusions, while 1-h post-vedolizumab infusion blood samples were drawn from the opposite arm also to determine peak levels. Also, during endoscopic assessments at week 6, biopsy specimens were obtained from 4 patients to assess for histological healing using the Robarts histological index (RHI).

The inclusion criteria were the following: aged between 18 and 80 years; male or female patients capable of giving voluntary informed consent; diagnosis of moderate-to-severe active UC and CD (Mayo endoscopic subscore > 1 for UC and presence of frank ulcerations for CD); and patients that were naïve to vedolizumab. The exclusion criteria were the following: signs of abdominal abscess; any colonic resection; either total or subtotal colectomy; known fixed stenosis of the intestine, ileostomy, or colostomy, use of non-permitted IBD therapies within 30 or 60 days; active or dormant TB infection; hepatitis C or B chronic infection; malignancy; small or large bowel malignancy; being incapable of complying with study protocols or attending study visits.

### Vedolizumab trough and peak level measurements

Vedolizumab serum levels were assessed using a vedolizumab enzyme-linked immunosorbent assay (ELISA) (KRISHGEN Biosystems, INDIA). The lower limit of detection was 0.028 µg/ml, and the percentage of coefficient of variation (%CV) was < 10% in the lower concentrations (32–125 µg/ml) and < 5% in the higher concentrations (> 250 µg/ml). These levels were obtained at each study visit, at weeks 2 and 6, before receiving vedolizumab infusions and post-infusions (weeks 0, 2, and 6) to determine peak vedolizumab levels.

### Independent covariates and Vedolizumab clinical response

Baseline and follow-up clinical response covariates were evaluated, including baseline age, weight, anti-TNF-α therapy history, serum albumin concentration, serum C-reactive protein (CRP) level, fecal calprotectin concentration, and the effect of prior immunosuppressants or corticosteroid therapy.

### Outcome measures

Baseline and follow-up clinical activity was carried out by employing the Crohn’s disease activity index (CDAI), and Harvey Bradshaw index (HBI) for CD and the partial Mayo score (PMS) for UC at weeks 0, 2, 6, and 14. The following outcomes were employed to investigate response:

1. For UC, clinical response was characterized by total Mayo score reduction of ≥ 3 points and ≥ 30% from baseline, reduction in bleeding per rectum subscore of ≥ 1 point or absolute bleeding per rectum subscore of ≤ 1, and CDAI reduction of > 70 points and HBI ≤ 3 points for CD.
2. For UC, clinical remission was characterized by a complete Mayo score of ≤ 2 points and no subscore > 1; for CD it was defined as a CDAI score < 150, and HBI score < 4.
3. A Mayo endoscopic subscore of ≤ 1 point determined an endoscopic response for UC, and an SES-CD score ≤ 2 determined the endoscopic response for CD.
4. Histological healing was defined as an RHI score < 6.

Early therapeutic drug trough levels at weeks 2 and 6, and clinical outcomes at week 14 were investigated using clinical remission as the sole outcome measure. At the end of induction (week 6), non-response was determined if patients failed to demonstrate response or there was a worsening of disease activity by week 14. Patients were considered primary non-responders in the presence of adequate trough level (> 8 µg/ml).

### Statistical analysis

Median, 95% confidence interval or mean, and standard deviation (SD) were used to represent continuous variables. The Mann–Whitney U test was utilized for continuous variables to compare baseline patient characteristics data and the clinical response of patients at various time intervals. Also, the Pearson chi-squared test was applied for the comparison of categorical data. The Wilcoxon rank test was used to evaluate the variation in vedolizumab levels during the study period. The Spearman rank test was incorporated to assess the correlation. Using logistic regression analysis, the strength of the correlation between clinical parameters and clinical response was analyzed at the end of the induction period. The best cut-off value of vedolizumab concentration for week 6 and week 14 clinical remission was anticipated using a receiver operating characteristic (ROC), and the value of high sensitivity and low specificity was chosen. Finally, the difference between quartiles was tested using a one-way analysis of variance (ANOVA). The SPSS Statistical Software, version 28 (IBM Chicago, USA), was used to conduct the analysis. A two-sided *P* value of < 0.05 determining statistical significance.

### Ethical approval

This study was approved by the Institutional Ethical Committee of King Abdulaziz University Hospital (480-20, 2020). The study design conforms to the 1975 Declaration of Helsinki.

## Results

### Patient demographics and clinical outcomes

**Figure 1:**
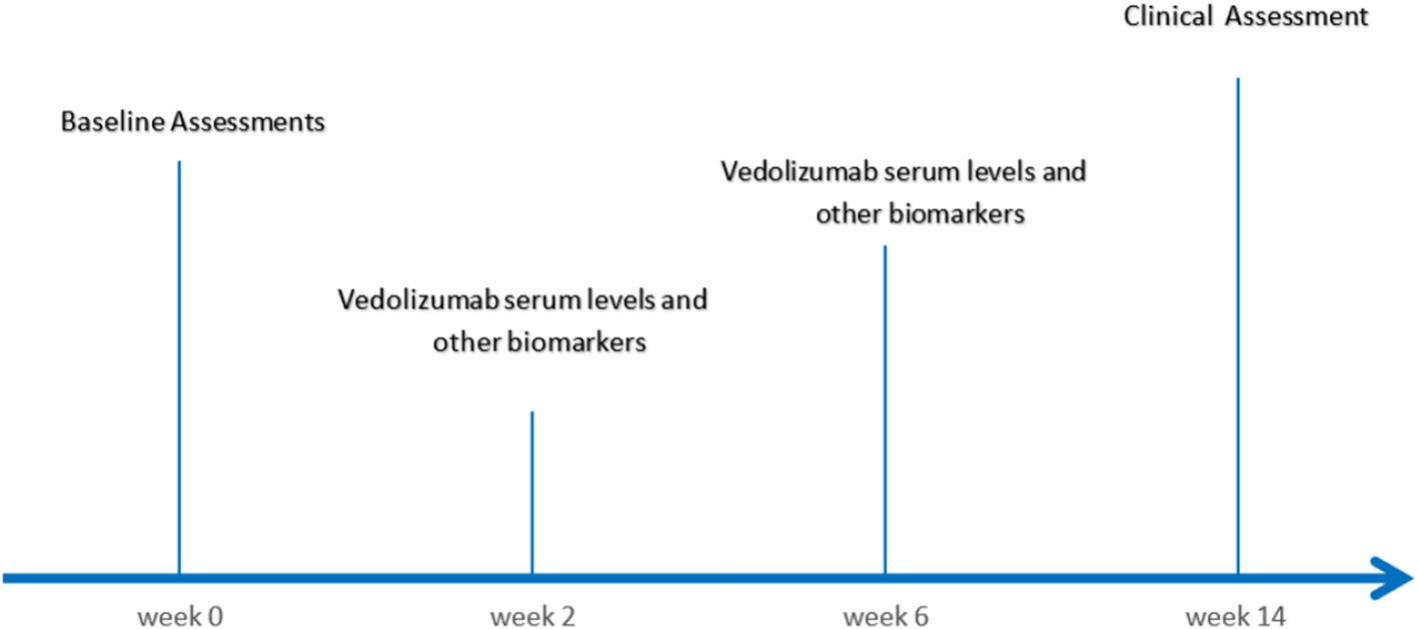
Patient characteristics and blood sample collection scheme.

In the study, 16 IBD patients (12 UC, 4 CD) commenced with vedolizumab therapy and were observed following completion of the induction period until week 14. Table 1 displays patient demographics and baseline clinical features, and the patient sample collection timeline is shown in Fig. 1.

**Table 1:** Clinical characteristics and patient demographics at baseline.

	Overall	UC	CD	*p* value
N (%)	16	12 (75)	4(25)	ND
Age (year)	30 ± 11.3	31.8 ± 11.6	25 ± 10.2	0.17
Malen(K)	7 (43.8)	4 (33.3)	3(75)	0.14
Female n {%}	9 (56.3)	a (66.7)	1(25)	ND
BMI	22.1 ± 3.6	22.7 ± 3.5	20.5 ± 3.8	0.33
Complete Mayo score		4.2 ± 2.8		ND
CDAI			272.5 ± 223.9	ND
Disease duration (Month)	45,157	39,8 ± 54.3	63 ± 26.6	0.06
Fecal calprotectin (ug/g)	2031 ± 3140.9	2260.5 ± 3431.9	1001.9 ± 1353.4	0.31
Albumin (g/L)	39 ± 7.9	41.1 ± 4,5	33.1 ± 13.6	0.26
CRP(mg/L)	11.3tl7.5	7.2 ± 6	32.7 ± 35.5	0.15
Hemoglobin (g/L)	11.8 ± 2.6	11.8 ± 2	10.4 ± 4.1	0.29
Naive (%)	14 (87.5)	12 (100)	2(50)	0.01
Pervious failure to Anti-TNF (%)	2 (12.5)	0 (0.0)	2(50)	ND
Concomitant immunomodulator (%]	3 (18.S)	3(25)	0 (0.0)	0.16
Concomitant steroid (%)	5(31.3)	4(33.3)	1(25)	0.75

*ANTI-TNF* Anti-tumor necrosis factor-α, *N* Number of patients, *UC* Ulcerative colitis, *CD* Crohn’s disease, mean (standard deviation), *ND* Not determined.

At the end of week 6, 14 patients had responded to vedolizumab therapy (82%), of which, 12 patients (86%) had UC, and two patients (14.3%) had CD. At week 14, vedolizumab responder rate was 9/16 (52.9%), 8 (66.6%) were UC patients, while 1 (25%) patient was CD. Seven patients (43%) were non-responders at the end of the induction period, and 4 of these patients (57.1%) had UC, and 3 (42.9%) had CD. Only 2 patients were considered as primary non-responders. Also, 47.1% of patients experienced steroid-free remission.

In total, 7 (37.5%) patients had available data on endoscopic remission at week 14; 4 patients (25%) achieved endoscopic remission based on Mayo endoscopic score, and 2 UC patients achieved histological healing according to RHI scoring with a mean week six vedolizumab trough level of (42.3 µg/ml ± 24.3) compared to the remaining two which did not show histological healing, one patient was UC (RHI = 22) and the other was CD (RHI = 17) which had a mean week six vedolizumab trough level of (30 µg/ml ± 14.4).

### Covariates association with vedolizumab trough level and clinical response

The median vedolizumab trough levels were higher among females than males at week 2 (30.1 ± 12.1 vs. 12.5 ± 11.3) and week 6 (25.1 ± 19.6 vs. 15.1 ± 33.5). Moreover, Sex correlated significantly with week 2 and week 6 vedolizumab trough levels in univariate analysis (*P* = 0.02). The male mean weight and BMI with the mean trough and peak vedolizumab levels showed an insignificant difference from the corresponding values in females (Table 2). However, 7 (43.8%) females demonstrated a meaningful clinical response at week 14 compared to 2 (12.5%) males (p = 0.04). Albumin levels showed no significant correlation with vedolizumab trough levels at week 2 *(P* = 0.33) and week 6 (*P* = 0.77). BMI was the only covariate with a significant association with week 6 vedolizumab serum trough levels (*P* = 0.02) based on the multivariate analysis (Table 3). There was no difference in the association between albumin levels (*P* = 1.0) and CRP (*P* = 0.70) with the clinical response at week 6, or between albumin levels (*P* = 0.68) and CRP (*P* = 0.11) and clinical response at week 14. Hemoglobin had a statistically significant relationship with the clinical response at week 6 (*P* = 0.03) but not at week 14 (*P* = 0.60). At week 6, there was a significant association between IBD type and clinical response (*P* = 0.01), but not at week 14 (*P* = 0.14). Prior use of immunomodulator therapy and Sex showed a significant difference among clinical responders at week 14 only (*P* = 0.046 and *P* = 0.045), respectively.

**Table 2:** Comparison of mean values between enrolled male and female patients.

	N (%)	Weight	Age	WK2		WKG	
				Peak level	Trough level	Peak level	Trough level
Male	7 (43.8}	68 + 8.5	31.5 ± 15.6	127.3 ± 44	54.8 ± 63.6	164.4 ± 61.6	10.4 ± 4.5
Female	9 (56.3}	51.6 + 9.8	29 ± 7.5	313.2 ± 195.8	44 ± 48.2	162.1 ± 71.6	27.5 ± 19.6
*p* value	ND	0.7 S	0.05	0.58	0 53	0.09	1.0

**Table 3:** Univariate and Multivariate Analysis of clinical parameters associated with week 2 and week 6 Vedolizumab trough levels.

Clinical parameters	WK 2		WK 6	
	Univariate	Multivariate	Univariate	Multivariate
Baseline Albumin (g/LJ	0.33	0.77	0.77	0.49
Baseline CRP (mg/L)	0.97	0.30	0.55	0.43
Baseline Hb (g/L)	0.59	0.74	0.57	0.62
Baseline faecal calprotectin (mg/L)	0.35	0.12	0.55	0.51
BMI	0.18	0.47	0.20	0.02
SEX	0.02	0.18	0.03	0.17
Age (year)	0.23	0.27	0.65	0.33
IBD Type	0.71	0.33	0.39	0.77
Concomitant immunomc-dulator	0.31	0.20	0.84	0.77
Concomitant steroid	o.ss	0.90	0.77	0.59
Pervious Anti-TNF	0.75	0.13	1.0	0.09

*CRP* C-reactive proteins, *HB* Haemoglobin, *Anti-TNF* Anti-tumour necrosis factor.

### Correlation of vedolizumab peak and trough levels with the adjusted dose

Table 4 shows that body weight was highly variable, and upon adjusting the dose for each patient according to their weight, the results showed a highly variable dose in mg/kg, with a mean SD (range) of 5.3 ± 1.3 (3.5–8.9) mg/kg. However, a trend towards a positive linear correlation between the dose expressed as mg/kg and trough level at week 2 was r = 0.70 (*P* = 0.002), and the dose with trough levels at week 6 was r = 0.49 (*P* = 0.052). Also, it was discovered that there was a substantial positive association between dose mg/kg and peak vedolizumab level at week 2 (r = 0.71, *P* = 0.002), and week 6 (r = 0.69, *P* = 0.003).

**Table 4:** Correlation of adjusted dose of vedolizumab with its peak and trough levels.

Weight (kg)	BMI	Adjusted dose (mg/kg)	WK 2		WK 6	
			Peak level	Trough level	Peak level	Trough level
58.6	25.36	5.1	401.83	30.18	208.76	34.07
59.1	22.80	5.0	142.86	22	195.85	7.89
55.6	23.75	5.3	169.22	43.22	158.16	3.7
33.6	15.76	8.9	321.92	42.06	221.94	40.3
45.7	23.01	6.5	564.78	46.14	267.12	59.58
48.6	18.30	6.1	665.99	18.11	248.72	19.86
56.2	24.91	5.3	200.26	23.36	167.09	25.12
45	20.49	6.6	250	33.29	301.37	49.07
68.4	21.41	4.3	102.04	11.68	91.84	8.18
67.3	30.4S	4.4	112.24	7.59	141.58	7.79
63	16.13	4.7	181.12	39.14	213.01	13.43
84	23.18	3.5	117.35	12.58	125	15.19
63.9	19.63	4.9	164.54	16.49	62.5	8.76
60.3	23.148	4.9	62.5	8.67	44.93	4.09
75	21.66	4	164.54	14.6	102.04	16.36
62.6	24.52	4.7	89.29	5.84	60.16	7.59
Correlation of level and adjusted dose*			r = 0.71 *p* = 0.002	r = 0.70 *p* = 0.002	r = 0.69 *p* = 0.003	r = 0.49 *p* = 0.05
Correlation of level with BMI*			r = − 0.30 *p* = 0.24	r = − 0.31 *p* = 0.24	r = − 0.32 *p* = 0.22	r = − 0.30 *p* = 0.25
Correlation of level with weight*			r = − 0.56 *p* = 0.02	r = − 0.60 *p* = 0.01	r = − 0.57 *p* = 0.02	r = − 0.33 *p* = 0.20

*r* Spearman correlation coefficient.

*Type of statistical test.

### Vedolizumab serum trough levels during the induction phase and clinical outcome analysis

**Figure 2:**
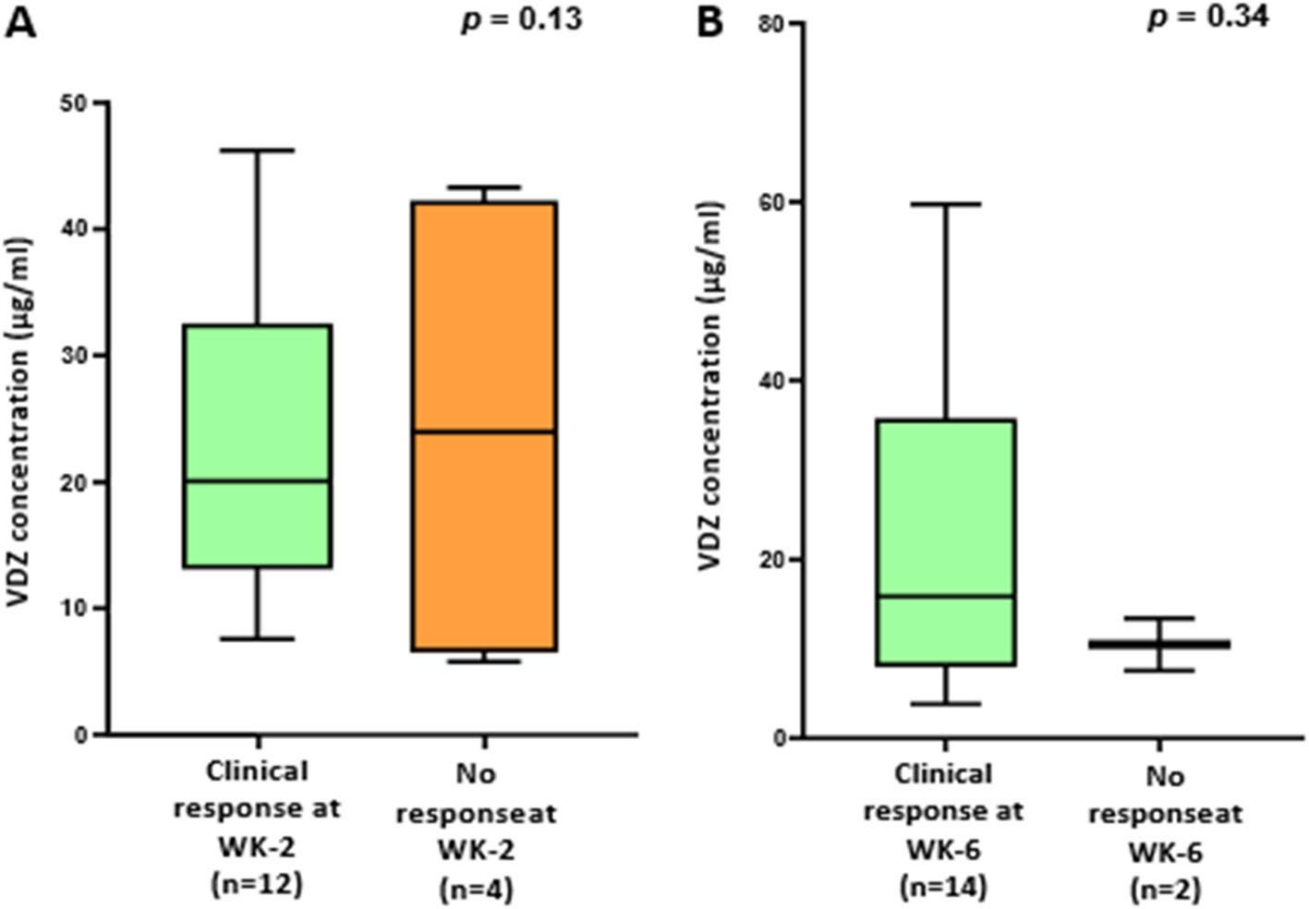
(**A**) A boxplot of Week 2 Vedolizumab trough levels in responders and non-responders. (**B**) A boxplot of trough levels of Vedolizumab in responders to Vedolizumab therapy compared to patients with no response at week 6. *P* value Mann–Whitney test.

**Figure 3:**
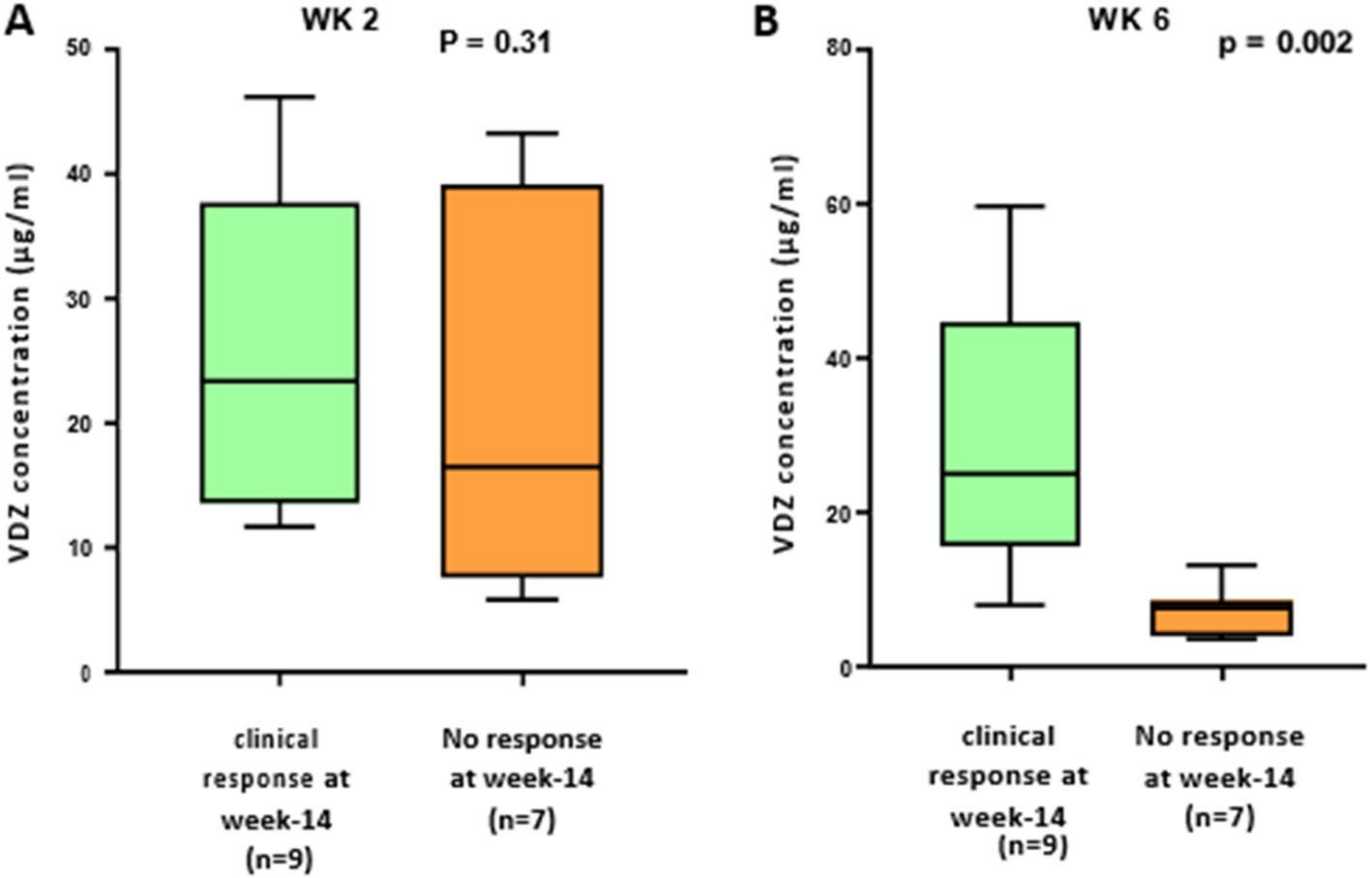
A box plot at week 14 showing the trough level of vedolizumab was categorized based on clinical response. (**A**) Patients with a response at week 14, along with patients with no response, were categorized based on their vedolizumab trough levels from week 2. (**B**) Patients with a response at week 14, along with patients with no response, were separated into two groups based on trough levels at week 6. *P* values Mann–Whitney test.

Week 2 responders had a higher median vedolizumab trough level concentration (27 µg/ml, 95% confidence interval (CI): 19.5–36.1) than non-responders (10.1 µg/ml, 95% CI: 4.1–16.2) (*P* = 0.13) (Fig. 2A). The median vedolizumab concentration showed a difference between responders (15.7 µg/ml, 95% CI: 11.1–31.7) and non-responders at week 6 (10.5 µg/ml, CI: 0–47.6), however, there was no noticeable difference overall (*P* = 0.34) (Fig. 2B). To assess week 14 clinical response based on vedolizumab trough levels, trough levels at weeks 2, and 6 were used to stratify week 14 clinical remission. At week 2, vedolizumab trough levels did not reach a significant level (*P* = 0.31), despite responders having a higher median trough level (23.3 g/ml, CI: 15.9–35.6) than non-responders (16.4 g/ml, CI: 6.2–34.5) (Fig. 3A). While at week 6 patients that responded to vedolizumab treatment had a significantly greater vedolizumab trough levels (25.1 µg/ml 95% CI: 16.5–42.9) than non-responders (7.7µg/ml, 95% CI: 4.6–10.6) (*P* = 0.002) (Fig. 3B). Furthermore, on multivariate analysis, the significance of vedolizumab trough levels for clinical remission at week 6 prediction remained substantial at week 14 (*P* = 0.01) (Tables 5, 6).

**Table 5:** Week 6 Clinical parameters univariate analysis association with week 14 clinical remission.

Clinical parameters	Median (WK 6)		*p* value	Median (WK 14)		*p* value
	Clinical remitters	Mori remitters		Clinical remitters	Non remitters	
WK 2 trough (μg/ml)	27	10.1	0.01	23.3	16.4	0.31
WK 6 trough (μg/ml)	15.7	10.5	0.34	25.1	7.7	0.002
Baseline CRP (mg/L)	5.3	ND	0.63	3.3	13.2	0.10
Baseline Albumin (g/L)	42.1	38.8	0.87	42.3	40.3	0.63
BMI	22.9	23.2	0.52	21.4	23.0	0.56
Age (year)	30	29	0.74	30	23	0.87
Disease duration	24	84	0.07	24	24	0.87

*CRP* C reactive proteins, *IBD* Inflammatory bowel disease, *ND* Not determined.

**Table 6:** Clinical parameters association with week 6 and 14 clinical remissions.

Clinical parameters	Multivariate analysis	
	WK 6 remission	WK 14 remission
	*p* value	*p* value
Vedolizumab level at week 6	0.53	0.01
Baseline CRP (mg/L)	0.60	0.18
Prior ariti-TNF therapy	0.78	0.37

*CRP* C reactive proteins, *anti-TNF* Anti-tumor necrosis factor.

We evaluated primary non-response patients, i.e., non-responders with adequate trough levels: only 2 of 7 non-responders had adequate trough levels at week 14. The only clinical parameter that exhibited a significant relationship in a regression analysis with vedolizumab trough levels was gender (OD: 11.6 95% CI: 0.012–1.1, *P* = 0.045); all other parameters showed no correlation.

We compared trough levels of vedolizumab at weeks 2 and 6 to identify if levels changed between each infusion and between other responders and non-responders: the results showed no statistically significant distinction in vedolizumab levels between week 2 and week 6 with responders (*P* = 0.69) and non-responders (*P* = 0.65).

### Vedolizumab serum peak levels in the induction phase and clinical outcome analysis

Week 2 responders has a higher median peak vedolizumab levels (190.6 µg/ml, 95% CI: 151–387) compared to non-responders (126.9 µg/ml, 95% CI: 36.9–203), however, the difference did not reach statistical significance (*P* = 0.058). Week 6 responders showed a slight difference in the median peak vedolizumab levels among responders (177 µg/ml, 95% CI: 119.7–214) than non-responders (134.5 µg/ml) (*P* = 0.70).

Week 14 clinical response was stratified by week 6 vedolizumab peak levels and resulted in no statistically significant difference among responders compared to non-responders (208.7 µg/ml, 95% CI: 101–239 vs. 158.1 µg/ml, 95% CI: 92–215) (*P* = 0.75).

### Quartile analysis of vedolizumab serum trough levels

**Figure 4:**
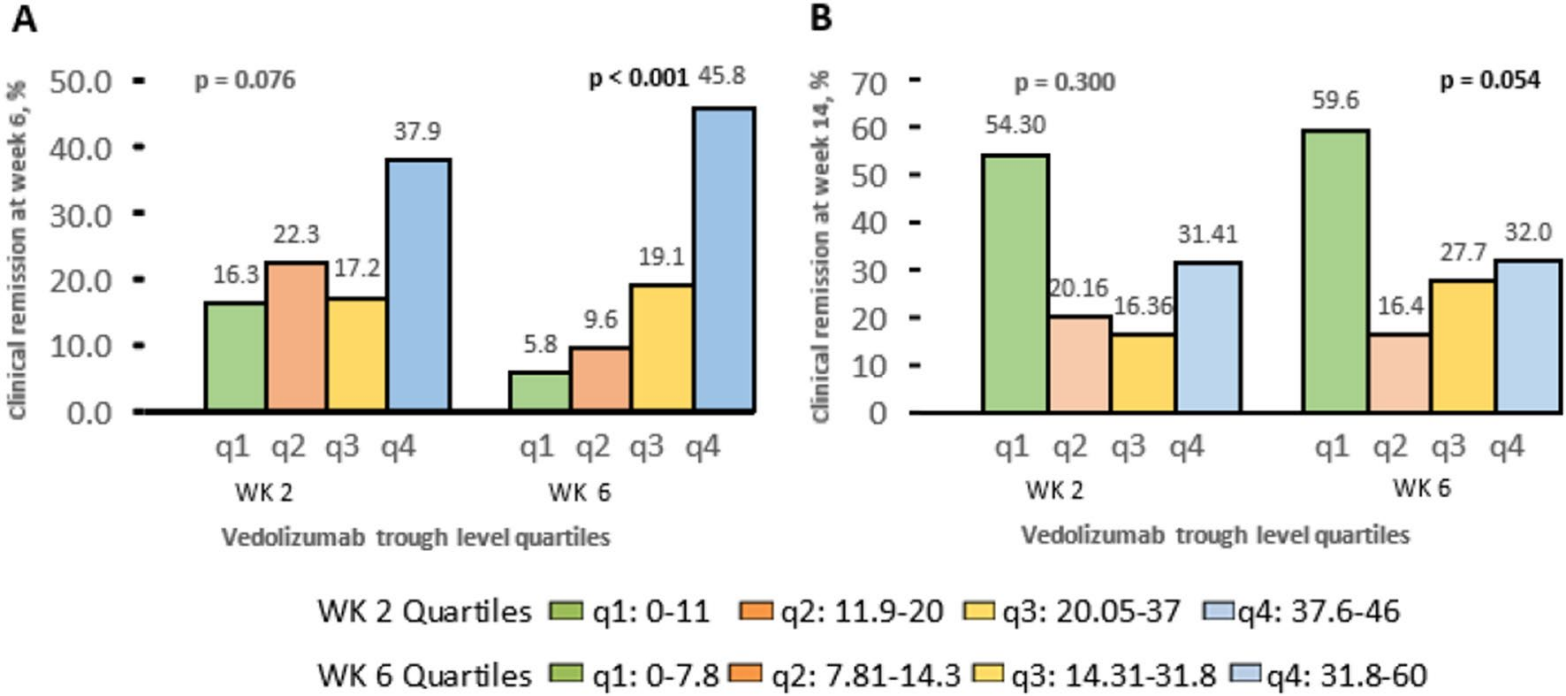
(**A**) Clinical remission was stratified at week 6 based on week 2 and week 6 vedolizumab trough level quartiles from the minimum (Q1) to the maximum quartile (Q4 (**B**) Clinical remission at week 14 classified according to vedolizumab trough levels quartiles at week 2 and week 6 from the minimum quartile (Q1) to the maximum quartile (Q4). *P* value, one way ANOVA.

The clinical outcomes according to quartiles of vedolizumab trough level were analyzed, and clinical remission at weeks 6 and 14 were classified according to quartiles of vedolizumab trough levels at weeks 2 and 6 (Fig. 4A, B). At week 6, vedolizumab trough level quartiles showed an important link between clinical remission and vedolizumab trough levels (*P* < 0.001). We observed an insignificant association between trough levels at week 6 and clinical remission at week 14 (*P* = 0.054).

### Vedolizumab Serum trough level receiver operating characteristics

**Figure 5:**
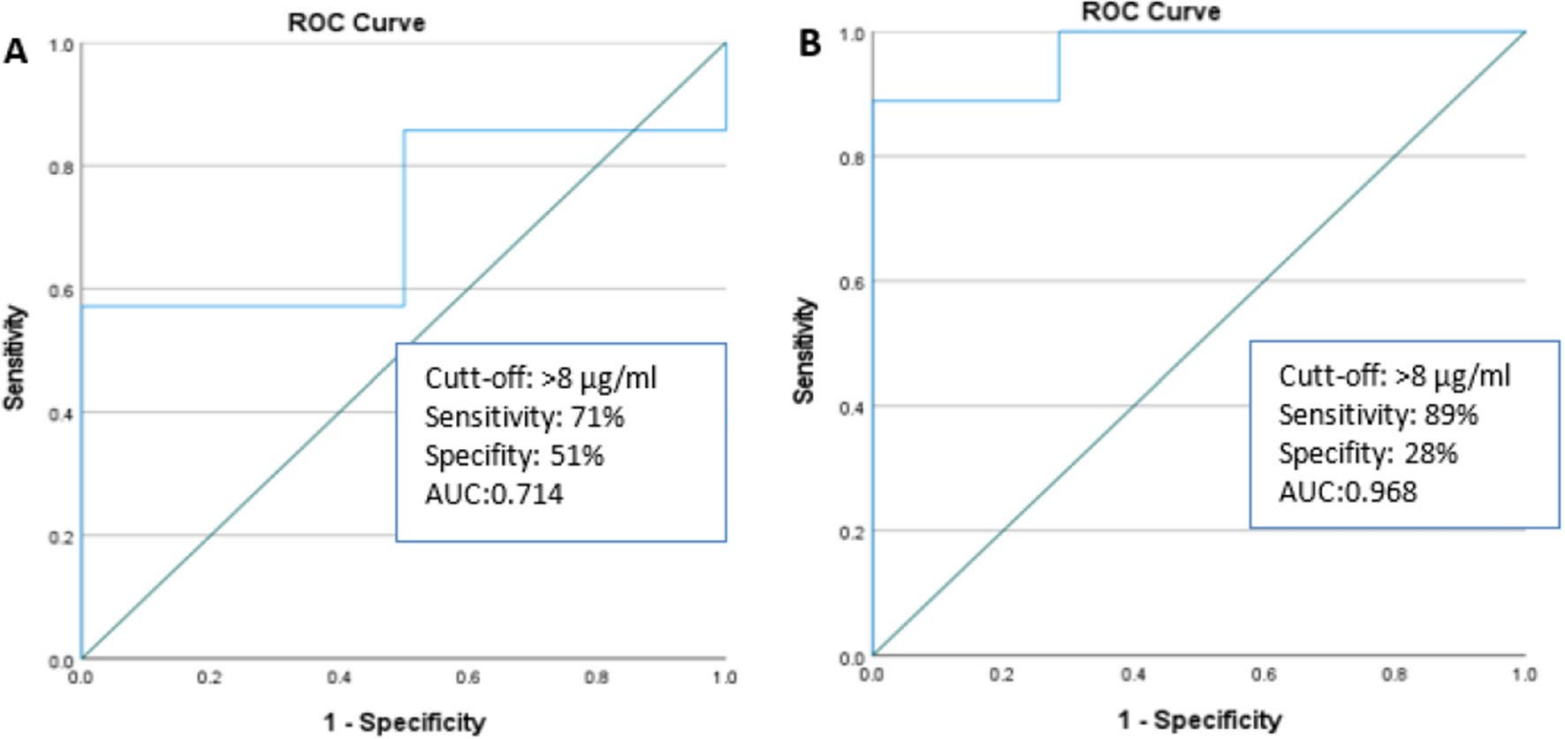
(**A**) ROC analysis of vedolizumab trough level concentration at week 6, clinical remission was the dependent variable (**B**) At week 14, ROC analysis was performed with clinical remission as the dependent variable.

A receiver operating characteristics (ROC) analysis at week 6 indicated only a modest discriminatory accuracy (AUC, 0.714; 71% sensitivity, 51% specificity for vedolizumab levels of > 8.00 µg/ml, *P* = 0.341). The ROC analysis at week 14 showed a high discriminatory accuracy (AUC, 0.968; 89% sensitivity, 28% specificity for vedolizumab levels of > 8.00 µg/ml, *P* = 0.002). At week 6, a cut-off value of > 8 µg/ml vedolizumab trough levels in patients with IBD predicted short-term clinical response at week 14 (Fig. 5).

## Discussion and conclusions

In a number of previous clinical studies, evidence has been provided showing the efficacy of vedolizumab in treating moderate-to-severe IBD^9–11,21–23^; however, non-response to vedolizumab therapy has been a significant issue that impacts patient health and causes a considerable burden on governments and economies, usually through direct or indirect costs^24^. Thus, in the current study, we aimed to identify whether early vedolizumab trough levels could predict patient response at week 14. The results showed that clinical remission at week 14 can be predicted by early analysis of vedolizumab trough levels at week 6. Indeed, with the present cohort, it was shown that early vedolizumab trough level measurements could be beneficial for obtaining indicative levels during the induction phase and providing favorable clinical outcomes. These findings back up prior research that shows that early trough level assessment can indicate response outcomes. A post hoc analysis by Osterman et al.^25^ of GEMINI trials investigated vedolizumab exposure–response relationships in UC patients. The study concluded that vedolizumab concentration proposed targets at weeks 6 and 14 was 37.1 µg/ml, 18.4 µg/ml, respectively, and 12.7 µg/ml at a steady state. Also, the study discovered that to determine weeks 14 and 52 clinical remission, week 6 was the earliest period that showed predictive vedolizumab trough level measurements. Several other cohort studies have found that early vedolizumab concentration improved clinical outcomes. A retrospective study by Dreesen et al. involving 179 patients from Belgium showed that vedolizumab concentrations in UC and CD at weeks 2 and 6 had levels > 30 µg/ml and > 24 µg/ml respectively, and > 14 µg/ml of vedolizumab throughout maintenance treatment were connected to enhanced efficacy^16^. Also, in a recent study by Guidi et al. an evaluation of week 14 vedolizumab trough levels was shown to be related to increased likelihood of maintaining vedolizumab in the first year of treatment^22^.

In clinical practice, there is often thought to be inadequate evidence to support using therapeutic drug monitoring (TDM)-guided optimization strategies for non-responsive patients with IBD. However, according to Dreesen et al.^12^, early vedolizumab trough level assessment may assist physicians in distinguishing between two groups of patients: those that have subtherapeutic levels and potentially benefit from dose adjustments which they might have experienced insufficient exposure or high vedolizumab clearance and those that remained non-responsive despite adequate therapeutic levels owing to mechanical failure or insufficient time^16^. TDM in vedolizumab has not been used for routine practice because the small quantity of vedolizumab considered sufficient to saturate cell α4β7 receptors contradicts the drug exposure–efficacy hypothesis^6^. However, several studies have shown variation in vedolizumab cut-off values for predicting clinical exposure with vedolizumab response in the induction period. Yacoub et al.^26^ conducted prospective research in 3 French referral hospitals to monitor trough levels during induction therapy with vedolizumab. In 82 IBD patients, median vedolizumab trough levels at week 2 were 27 µg/ml, at week 6 was 23 µg/ml, and at week 14 was 10.7 µg/ml, with a cut-off of > 18 µg/ml at week 6 and showing mucosal healing within 54 weeks of therapy. Another trial by Williet et al. that included 47 IBD patients found that patients within the first six months that had vedolizumab trough levels of < 18.5 g/ml necessitated continuous therapy (100% positive predictive value, 46.2% negative predictive value; AUC, 0.72)^27^. Moreover, a recent study suggested even higher vedolizumab trough levels at week 6 (> 28µg/ml) with an AUC of 0.723 (95% confidence interval = 0.567–0.878, *P* = 0.02), indicating a persistent response during the follow-up period, which lasted an average of 11 months^28^.

The median vedolizumab trough levels in our study at week 6 were lower than previously reported studies for the same duration of time; hence our proposed cut-off value (> 8 g/ml) was similarly lower. Several factors could explain the low cut-off values found. Indeed, at the outset, the ELISA kit used in this study was different from the kit used in previous investigations, with a lower limit of detection (0.028 µg/ml), which could have led to the lower cut-off value determination. Also, the purpose of establishing cut-off values differed across the studies: in one study, it was used to identify histological healing^26^, and in another, it was incorporated for individuals that require dose changes^27^; alternatively, cut-off values were utilized for observing response prediction over one year^28^. However, the present cut-off values at week 6 indicate only short-term clinical response (week 14). Our cut-off value was determined using the optimal point of both high sensitivity and low specificity. Another reason for the present low values is the small sample size (n = 16), which was lower than in previous studies. Unlike other studies, we included Saudi Arabian nationals, which may have specific genetic backgrounds that may have affected trough levels and thus cut-off values, as suggested by Barnes et al. which found that patients with varied racial, ethnic, and genetic backgrounds respond differently to biologic therapy^29^. A single-center study showed that CD patients carrying a specific genetic trait may not respond to biological therapy despite having greater rates of stricturing or penetrating illness, and UC patients with the same genetic trait do not respond to immunomodulators^30^. Another study found that Asian individuals had a considerably lower likelihood of receiving biological treatments than white patients^31^. We may be able to tailor therapy and get a step closer to the concept of customized medicine for patients with IBD by detecting variations in the development and characteristics of IBD in individuals of different races.

Our quartile analysis of trough levels at week 6 was associated with a low percentage of clinical remission in the same period, but it is not significantly affecting the remission at week 14, suggesting that the presence of other variables could affect the response including the serum level at week 14, and/or tissue levels of vedolizumab. We identified a correlation between baseline BMI and vedolizumab trough levels, which differed between males and females. Also, the dose adjusted per weight was correlated with vedolizumab trough levels at the induction period. These results may indicate that patients might achieve improved clinical outcomes if the dose of vedolizumab could be adjusted depending on the patient's weight, and height. Patients received vedolizumab IV infusion (300 mg), the mean dose (mg/kg) in males was (4.46 ± 0.50) compared to females (5.9 ± 1.3) (*P* = 0.62). Gender had no statistically significant correlation with vedolizumab serum trough levels. Moreover, no correlation with albumin levels or CRP has been identified in the present study. This may be because the included patients have broadly average values of albumin levels (median albumin level, 41.5 IQR: 34.1–43.3) and CRP (median CRP, 3.4 IQR: 3.2–14.5). The present results were similar to the population used in a pharmacokinetic study, which showed that female sex and body weight were found to be linked with increased clearance of vedolizumab^32^. However, the pharmacokinetics study suggests that an individual with a body weight of 120 kg and an albumin concentration of 4.0 g/dL has a 19% chance of experiencing > 25% higher clearance than a patient with a body weight of 70 kg and an identical albumin concentration. There may be some limitations to our study, such as the limited sample size and insufficient endoscopic results. Future cohort studies with a greater sample size are necessary to justify the therapeutic range associated with the cost-effective management of IBD patients.

In conclusion, we found that week 6 vedolizumab trough levels differed across responders and non-responders, and we also identified that early vedolizumab trough level measurements with a cut-off value of > 8 μg/ml anticipated clinical response at week 14. Moreover, we observed that BMI, and dosage (mg/kg) were correlated significantly with vedolizumab trough levels. These findings support the use of vedolizumab level measurements at an early stage of treatment with dose adjustments per weight to identify individuals that might benefit from long-term vedolizumab therapy. Future work should focus on full pharmacokinetic analysis, genetic variables, and other pharmacodynamic variables that have been found to be linked with vedolizumab response.

## Data Availability

Data that support the findings of this study are available from the corresponding author, upon request.
